# Immunogenicity of a killed bivalent whole cell oral cholera vaccine in forcibly displaced Myanmar nationals in Cox's Bazar, Bangladesh

**DOI:** 10.1371/journal.pntd.0007989

**Published:** 2020-03-16

**Authors:** Fahima Chowdhury, Taufiqur Rahman Bhuiyan, Afroza Akter, Md Saruar Bhuiyan, Ashraful Islam Khan, Motaher Hossain, Imam Tauheed, Tasnuva Ahmed, Shaumik Islam, Tanzeem Ahmed Rafique, Shah Alam Siddique, Nabila Binta Harun, Khaleda Islam, John D. Clemens, Firdausi Qadri

**Affiliations:** 1 Infectious Diseases Division, International Centre for Diarrhoeal Disease Research Bangladesh (icddr,b), Dhaka, Bangladesh; 2 Directorate General of Health Services, Ministry of Health and Family Welfare, Dhaka, Bangladesh; 3 UCLA Fielding School of Public Health, Los Angeles, California, United States of America; 4 Korea University School of Medicine, Seoul, South Korea; Lowell General Hospital, UNITED STATES

## Abstract

After the large influx of Rohingya nationals (termed Forcibly Displaced Myanmar National; FDMN) from Rakhine State of Myanmar to Cox’s Bazar in Bangladesh, it was apparent that outbreaks of cholera was very likely in this setting where people were living under adverse water and sanitation conditions. Large campaigns of oral cholera vaccine (OCV) were carried out as a preemptive measure to control cholera epidemics. The aim of the study was to evaluate the immune responses of healthy adults and children after administration of two doses of OCV at 14 days interval in FDMN population and compare with the response observed in Bangladeshi’s vaccinated earlier. A cross-sectional immunogenicity study was conducted among FDMNs of three age cohort; in adults (18+years; n = 83), in older children (6–17 years; n = 63) and in younger children (1–5 years; n = 80). Capillary blood was collected at three time points to measure vibriocidal antibodies using either plasma or dried blood spot (DBS) specimens. There was a significant increase of responder frequency of vibriocidal antibody titer at day 14 in all groups for *Vibrio cholerae* O1 (Ogawa/Inaba: adults-64%/64%, older children-70%/89% and younger children-51%/75%). There was no overall difference of vibriocidal antibody titer between FDMN and Bangladeshi population at baseline (p = 0.07–0.08) and at day 14, day 28 in all age groups for both serotypes. The seroconversion rate and geometric mean titer (GMT) of either serotype were comparable using both plasma and DBS specimens. These results showed that OCV is capable of inducing robust immune responses in adults and children among the FDMN population which is comparable to that seen in Bangladeshi participants in different age groups or that reported from other cholera endemic countries. Our results also suggest that the displaced population were exposed to *V*. *cholerae* prior to seeking shelter in Bangladesh.

## Author summary

Oral cholera vaccines (OCV) are now on the WHO stockpile and targeted for use for countries with outbreaks and epidemics but also for control of endemic cholera. In Bangladesh many studies have been carried out to assess the safety, immunogenicity as well as feasibility of vaccination in the endemic settings of the country. However, with the large recent influx of displaced Rohingya nationals from Myanmar (termed Forcibly Displace Myanmar National; FDMN), mass campaigns with OCV were conducted between October 2017-December 2018. However, no data is available of the previous exposure to cholera of this population, prior to their arrival in Bangladesh. An assessment of immunogenicity status of FDMN is needed to find out if OCV is able to elicit comparable immune response and whether the same dose regimen of OCV was immunogenic among the FDMNs. In this study, we have measured the immune responses to the OCV, Shanchol, in adults (18 years and above), older children (6–17 years), and younger children (1–5 years). The results of this study shows that the oral cholera vaccine capable of inducing an immune response in adults and children among this FDMN population and the responses were comparable to that seen in Bangladeshi participants in earlier studies.

## Introduction

The Rohingya nationals from Rakhine State of Myanmar have been displaced to Cox’s Bazar in Bangladesh from 1978 [1]. However the largest displacement was from August 2017, when nearly 700,000 people (termed Forcibly Displaced Myanmar National; FDMN) entered Myanmar-bordered-Cox’s Bazar area resulting in over 1 million of these people currently living in Bangladesh [2–4]. The government of Bangladesh and other National and international NGOs provided the displaced people with temporary shelters, tube wells, latrines, limited health care access and basic necessities of life, these people live in densely populated conditions in Cox’s Bazar with poor hygienic conditions with lack of adequate safe water and sanitation. These factors increase the risk of water borne diseases by the faecal oral routes [5].

The accessibility of published information on the occurrence of diarrhoeal diseases is limited in Myanmar and almost nonexistent in the Rakhine state. The surveillance data from the Yangon Regional Health Center suggest that the severe diarrhea rate is estimated to be 2.6–3.5 per 100,000 population and the mortality rate is 0.04–0.1 per 100,000 [6]. Another report published in 2015 showed that the detection rates of *V*. *cholerae* O1 in stools from patients with severe diarrhea were 23% in 2012 and 14% in 2013 respectively [6]. WHO states that 16 and 103 cholera cases in Myanmar in 2011 and 2015 respectively and around a 12% case fatality rate[7, 8]. The Advisory Commission on Rakhine State in Myanmar has been stated in a report that the Rohingya populations have limited access to health care, higher child mortality rate and immunization coverage is lowest than seen in other Myanmar nationals [9].

In Cox’s Bazar, overcrowding in the camps has led to high rates of communicable diseases. The commonly reported public health problems among the FDMNs are acute watery diarrhea, measles, acute respiratory infections, diphtheria, malaria, other infectious diseases as well as malnutrition [10]. Cholera is endemic in Bangladesh [11] and it is prevalent in Cox’s Bazar [12]. The aim of the study was to evaluate the immune responses to OCV among the healthy FDMNs in Bangladesh and to compare the immune response of the FDMN participants with data of the vibriocidal responses seen in adults and children in an earlier vaccination study carried out in the Mirpur urban area in Dhaka.

## Methods

### Ethical statement

This study was approved by the institutional review board (Research Review Committee and Ethical Review Committee) of icddr,b and also permission was obtained from the Directorate General of Health Services (DGHS) as well as Refugee Relief Repatriation Commission (RRRC) of Bangladesh. Written informed consent was obtained from the participants or from parents or guardian and assent was also obtained from participants who were 11 to 17 years of age prior to enrollment. As the FDMN participants do not understand Bangla language, we appointed an interpreter for all study participants for clarification of the study procedure and obtaining informed consent from them. The interpreters were local community people who could understand both Bangla as well as the Arakanese language which the FDMNs speak.

### Field Site

**Fig 1:**
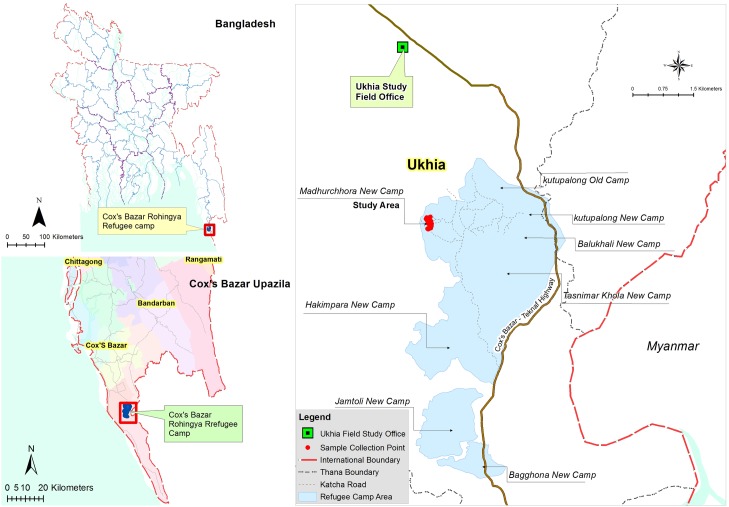
The study site in Ukhia (Map has been created by Arc GIS v 10.6).

The study was conducted at the Modhurchora Camp under Palong Khali Union of Ukhia upazilla in the Cox’s Bazar district. It is a remote area and with almost no access to transportation (Fig 1). The study area was hard to reach and was only accessible by foot. The camps of the study participants were 6–7 kilometers from the nearest health facility. The study population had also missed the first OCV campaign that was carried out in October 2017 [13] since they had arrived in Bangladesh after the vaccination had been carried out.

### Study participants

The study participants had recently been displaced to this area and most of them were from Muslim communities. Healthy males and females aged more than 1 year of age were enrolled.

Exclusion criteria included with chronic illness or any recent illness, currently on antimicrobial therapy, history of serious vaccine reaction, severe malnutrition, or diarrhea lasting for more than 2 weeks in the past 6 months. Participants having abdominal pain, fever more than 100.4^0^ F, vomiting in past 24 hours, any history of confirmed cholera and history of taking oral cholera vaccine or who were currently on antimicrobial therapy were excluded.

### Earlier Shanchol vaccination study in Bangladesh

We have conducted a clinical trial of OCV ‘Shanchol’ in Mirpur area of Dhaka city from July 2016 to May 2017 (ClinicalTrials.gov # NCT02823899). A total of 280 healthy participants of three age cohorts were given two doses of Shanchol 14 days apart. Blood was taken from the participants at three time points, prior to immunization on day 0 and day 14 and also on day 28. We have compared the immune responses of the FDMN study participants with the study participants as described above.

### Study design

This was a cross sectional study. The sample size was estimated based on the assumption of the antibody response in terms of *V*.*cholerae* O1 Ogawa: it was 72% for adults, 82% for older children and 75% for younger children. In this cross-sectional study with cohort follow up, the expected proportion of response to Shanchol with 10% precision and 5% level of significance, sample size was found to be 75 individual in the adult group, 57 individuals in the older children group and 72 individuals in the younger children group. With 10% attrition rate the inflated sample sizes was calculated to be 83 for adult, 63 for older children and 80 for younger children. Thus the total sample size was 226.

Study field staff and interpreters visited the different households in the study area and briefed the procedure to healthy participants and requested them to visit the field site in the camp on the study days for blood collection and vaccination.

### Study agent and administration

Shanchol (Shantha Biotechnics /Sanofi Pasteur) is a single dose vial vaccine containing 1.5 ml of heat killed and formalin killed whole cell bacteria *Vibrio cholerae* O1 and *V*. *cholerae* O139 as described previously [14]. Two doses of Shanchol (lot SCN011A16/SCN012A16) were administered, first dose at day 0 and 2^nd^ dose at day 14 among the participants. The vaccines were stored at +2–8°C in the EPI cold rooms. It was transported to the study site on the day of vaccination at ambient temperature [15]. The study staff shook the vial gently before administration of the vaccine to the participants to resuspend contents of the vaccine. After vaccination the participants were offered a half cup of water.

### Blood collection

Capillary blood specimens were collected in heparin capillary tubes before administration of the first dose of vaccine on day 0, on day 14 (before 2nd dose of vaccination) and day 28 by finger prick method. Blood collection was performed by trained laboratory assistant at the field site in the camp. Approximately 50 μl of capillary blood was adsorbed on each spot of the DBS paper to obtain dried blood spot (DBS card: Whatman 903, GE Healthcare) and air dried. After four to five spots were obtained, blood was collected in capillary tubes to separate plasma specimens for adults and older children. For younger children, we did not process blood in DBS cards. The dried DBS cards were placed in a single, gas-impermeable zipper bag containing desiccant sachets (2 desiccants per bag) to protect them from moisture. The DBS cards were kept at 2–8°C and plasma were then stored at -20°C in the field office in Ukhia in Cox’s Bazar. All DBS cards and plasma samples were transported within a week to the icddr,b laboratory in Dhaka and stored at -80°C.

### DBS Eluates

A 3.2 mm spot was punched with a DBS puncher (PerkinElmer, USA) from each blood-soaked circle of the DBS card. Punched spots from each participant were transferred to an eppendorf tube containing phosphate buffered saline (PBS, 10 mM, pH 7.2 with 0.05% bovine serum albumin) was added according to the desired dilution (18 punch in 270 μl; 1:10 dilution) for vibriocidal assay and kept at room temperature overnight. The tube was centrifuged (2500 x g for 7 min) and eluate was collected in a fresh eppendorf tube and used for immunological assays.

### Vibriocidal assay

Vibriocidal antibody titer was measured according to a procedure described recently [16]. Briefly, 25 μl of cold saline (0.154 M) was dispensed in all wells except column #2 of 96 well ELISA plates (Nunc F; Finland). Plasma and DBS eluates were heat inactivated at 56°C for 30 min. Plasma (1:10 diluted; 45 μl of cold saline and 5 μl of plasma) and 50 μl of DBS eluates were dispensed in corresponding well in column #2. The specimens two fold diluted in serial wells. The dilution was accomplished by mixing the solution in column #2, aspirating 25 μl and dispensing and mixing the sample in column #3 and so on, until column #12 (this equals to 1:10240). At the end, 25 μl was discarded from the last well in each row. *V*. *cholerae* O1 strain Ogawa (X25049) and Inaba (T19479) was cultured on blood agar plates at 37°C overnight. A loopful of bacteria was inoculated in Brain Heart Infusion (BHI) media for 2–3 hours at 37°C with shaking targeting OD at 0.4. Bacterial culture was centrifuged and resuspended in sterile saline and adjusted OD at 0.3 at 600 nm. For serum vibriocidal assay, 25 μl of bacteria (21.25 μl normal saline, 1.25 μl bacteria and 2.5 μl guinea pig sera) was added to all wells except wells in column #E1, F1, G1 and H1 of 96 well plates and 25 μl of cold saline added to wells # E1, F1, G1 and H1. For DBS vibriocidal assay, 25 μl of bacteria (21.25 μl normal saline, 1.25 μl bacteria and 2.5 μl guinea pig sera) was added to all wells except wells row #A2-A12, B2-B12 wells and column #E1, F1, G1 & H1 wells and 25 μl of cold saline added to wells # E1, F1, G1 and H1. The OD value of wells in row #A2-A12, B2-B12 was used as the colour background control for DBS. The plates were incubated at 37°C for 1 hour and 150 μl of BHI media was added to all wells and incubated again at 37°C. The plates were read spectrophotometrically (Eon, BioTek, Vermont, USA) and the absorbance for four control wells was considered positive if OD was between 0.20 to 0.28 at 595 nm (S1 Text) [17].

### Statistical analyses

We assessed differences in the magnitude of the responses using the Wilcoxon Signed Rank test for Paired samples and the Mann–Whitney U-test. Statistical analyses were performed using GraphPad prism software version 6 (GraphPad Software, Inc., La Jolla CA, USA). Two-sided P values <0.05 were considered significant. The Bland-Altman plots with 95% limits of agreement (LoA) were used to evaluate the agreement between results obtained with plasma and DBS. The 95% LoA was calculated as the average difference between the plasma and DBS ± 1.96SD (upper limit and lower limit). For comparing the GM among between FDMNs and Bangladeshi populations among different age groups, we calculated 95% confidence interval (CI) for each GM and if two CI did not overlap then we assumed that the GMs are significantly different from each other. Figures were generated in GraphPad prism.

## Results

### Study participants

We enrolled total 226 participants in February 2018 and followed them on day 14 and day 28 following vaccination. The three age cohorts were enrolled with 83 individuals in the adult group (18+years), 63 individuals in the older children group (6–17 years) and 80 individuals in the younger children group (1–5 years).

**Fig 2:**
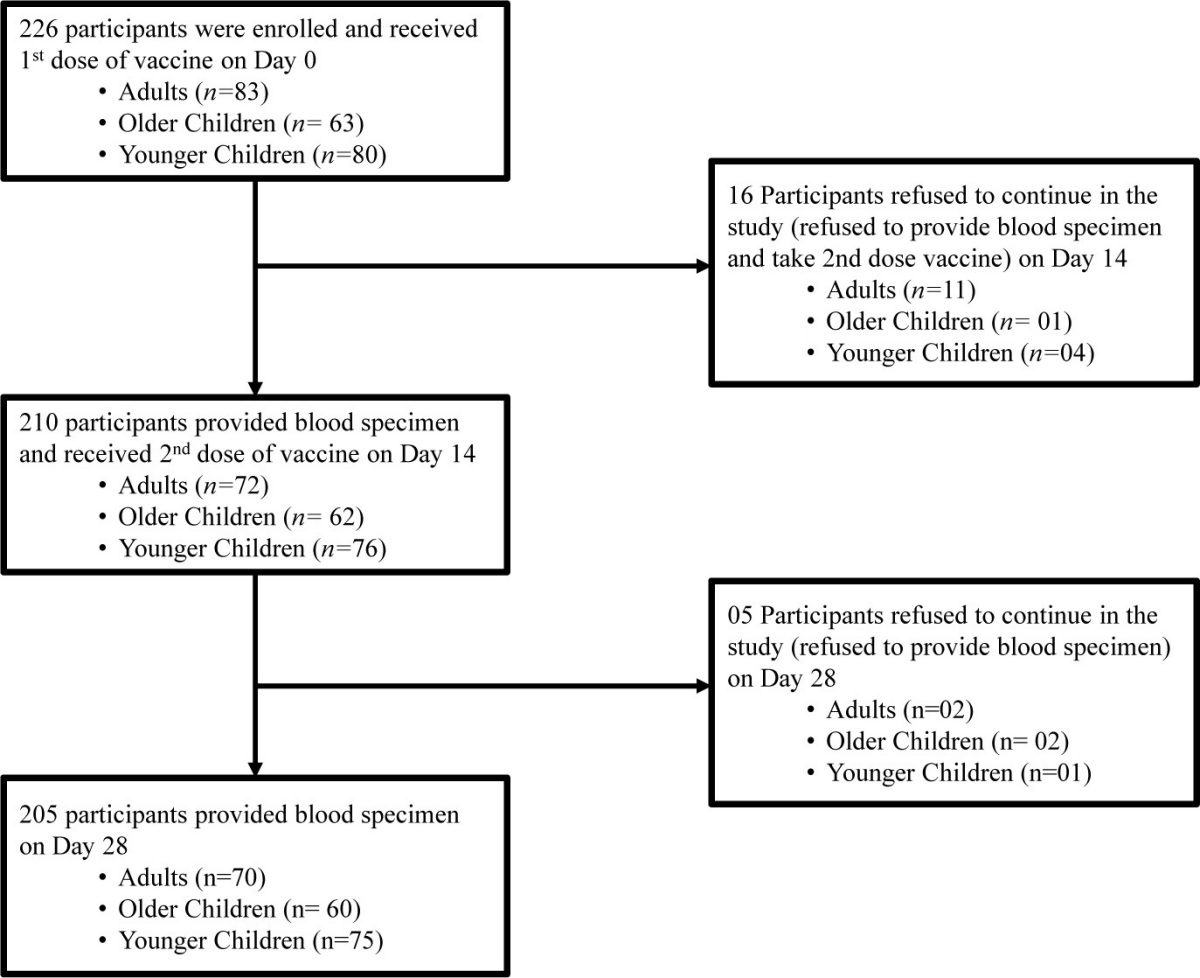
Flowchart of participants in the study.

The enrolment status of participants is shown in the study flow diagram (Fig 2).

Among 226 participants, 83 were adults (M:F = 36:47); 63 were older children (M:F = 30:33) and 80 were younger children (M:F = 43:37) (Table 1). The median age of the adult cohort was 27 years; older children was 10 years and for younger children it was 3 years. 21 participants were lost to follow up from the study (n = 13 for adults; n = 3 for older children and n = 5 for younger children). There was no significant difference in sex distributions in any of the three age groups (Table 1).

**Table 1:** Characteristics of study participants.

Characteristics	Adult (18 yrs and above)	Older Children (6 to 17 yrs)	Younger Children (1 to 5 yrs)
Male, no./total (%)	36/83 (43)	30/63 (48)	43/80 (54)
Female, no./total (%)	47/83 (57)	33/63 (52)	37/80 (46)
Age, mean (SD) years	32 (12.59)	10 (2.86)	3 (1.32)
Age, median years	27 (18,65)	10 (6,17)	3 (1,5)

### Vibriocidal antibody responses in plasma

**Fig 3:**
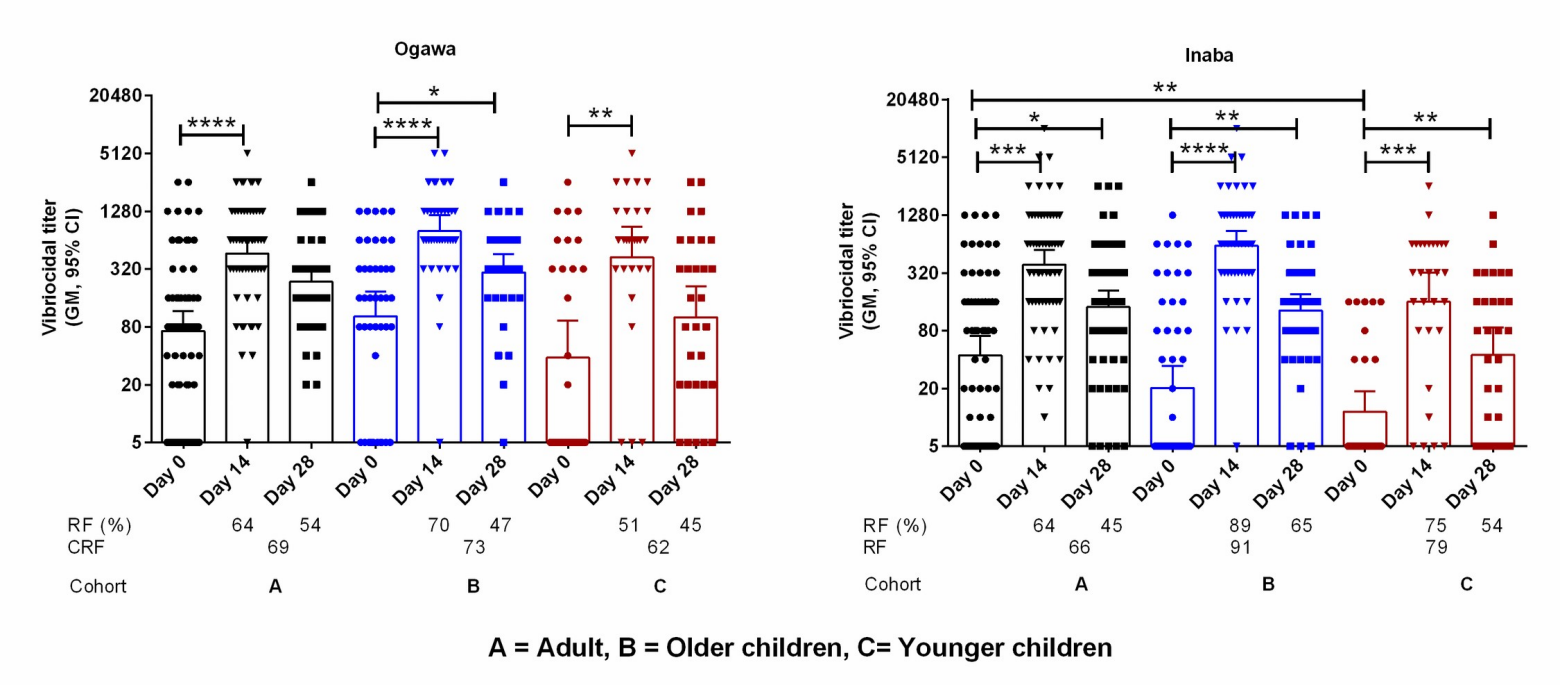
Vibriocidal antibody titer in the plasma of FDMN population to Ogawa and Inaba serotype in adult, older children and younger children. Asterisk denote the level of significance (*, P<0.05; **, P<0.01; ***, P<0.001 and ****, P<0.0001. Responder frequency (R/F) was calculated as four-fold rise of antibody titer compared to day 0 and cumulative responder frequency (CRF) calculated from either four-fold increase in any day post immunization (day 14 and day 28).

We assessed vibriocidal antibody responses in plasma specimens obtained from the FDMNs (Fig 3). There was a significant increase of responder frequency of vibriocidal titer at day 14 in all age groups both for Ogawa and Inaba (Ogawa/Inaba: adults-64%/64%, older children-70%/89% and younger children-51%/75%). At day 28, significantly increased vibriocidal antibody responses were observed for all age groups in comparison to the baseline vibriocidal antibody responses (Ogawa/Inaba: adults 54%/45%, older children 47%/65% and younger children 45%/54%).

### Vibriocidal antibody responses in DBS eluates

**Fig 4:**
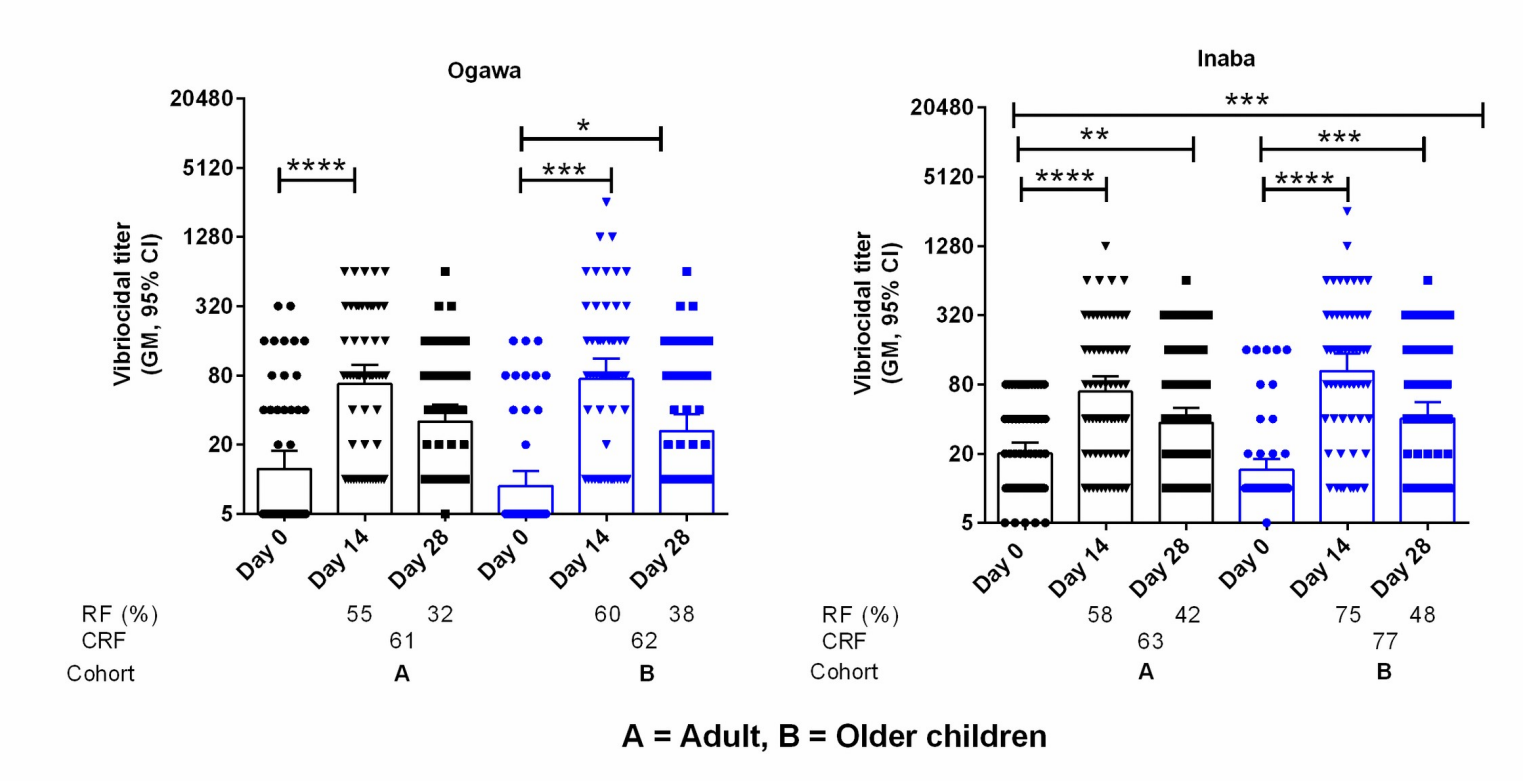
Vibriocidal antibody titer in the DBS eluates of FDMN population to Ogawa and Inaba serotype in adult and older children. Asterisk denote the level of significance (*, P<0.05; **, P<0.01; ***, P<0.001 and ****, P<0.0001. Responder frequency (R/F) was calculated as four-fold rise of antibody titer compared to day 0 and cumulative responder frequency (CRF) calculated from either four-fold increase in any day post immunization (day 14 and day 28).

We assessed vibriocidal antibody titer on day 0, day 14 and day 28 using DBS eluates collected from FDMN population against *V*. *cholerae* O1 Ogawa and Inaba (Fig 4). There was a significant increase of responder frequency of vibriocidal antibody titer at day 14 in the adult and older children groups (Ogawa/Inaba: adults-55%/58% and older children-60%/75%). Vibriocidal antibody responses were remained elevated on day 28 in adult and older children groups (Ogawa/Inaba: adults-32%/42% and older children-38%/48%).

### Comparison of vibriocidal antibody responses between DBS eluates and plasma

**Fig 5:**
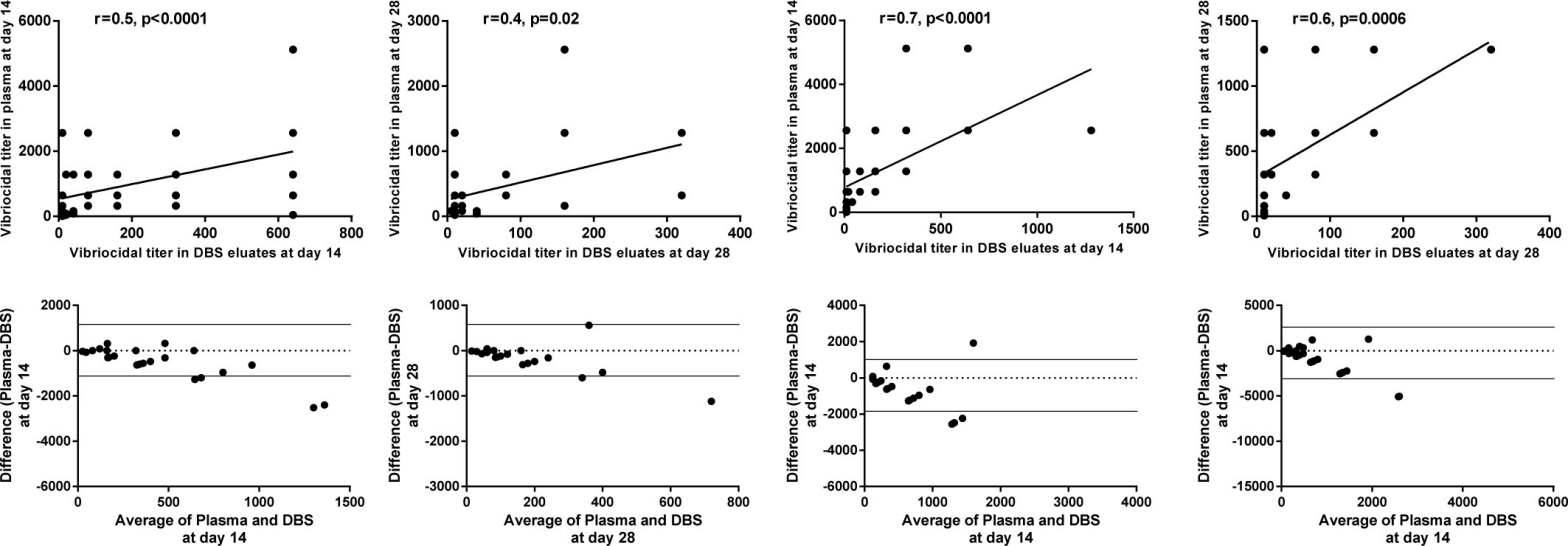
Correlations and the Bland-Altman analysis of vibriocidal antibodies in plasma and DBS eluates to Ogawa serotype in adults and older children. Spearman correlation of vibriocidal responses in plasma and DBS eluates at day 14 and day 28. Bland-Altman analysis of vibriocidal responses in plasma and DBS eluates of OCV recipients of FDMN at day 14 and day 28.

**Fig 6:**
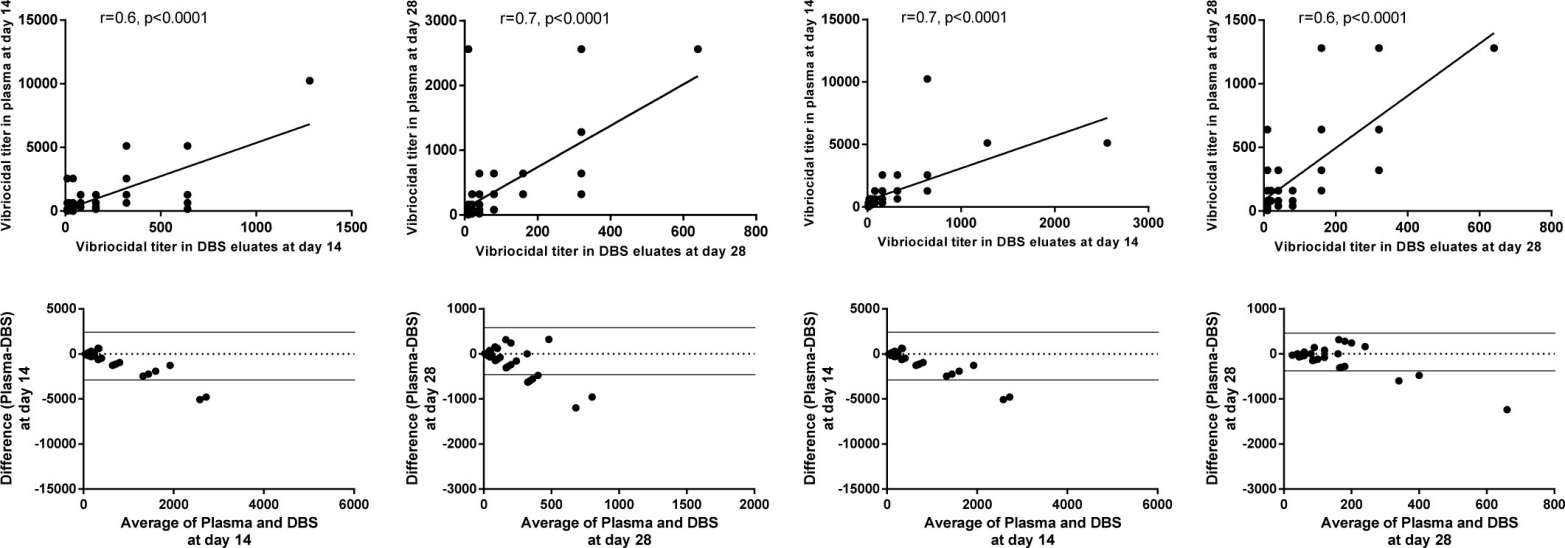
Correlations and the Bland-Altman analysis of vibriocidal antibodies in plasma and DBS eluates to Inaba serotype in adults and older children. Spearman correlation of vibriocidal responses in plasma and DBS eluates at day 14 and 28. Bland-Altman analysis of vibriocidal responses in plasma and DBS eluates of OCV recipients of FDMN at day 14 and 28.

We compared the vibriocidal antibody responses in DBS eluates with results obtained from the corresponding plasma specimens. There was significant correlation in results obtained between DBS eluates and plasma specimens at day 14 among adults (r = 0.5, p<0.0001) and older children (r = 0.7, p<0.0001) (Figs 5 and 6) to both *V*. *cholerae* O1 serotypes. We also analyzed the agreement of vibriocidal titer between DBS eluates and plasma by the Bland-Altman agreement analysis and observed good agreement of vibriocidal titer at day 14 (Figs 5 and 6). Similar agreement was also observed at day 28.

### Evaluation of vibriocidal antibody responses between the FDMN and Bangladeshi population after oral cholera vaccination

**Fig 7:**
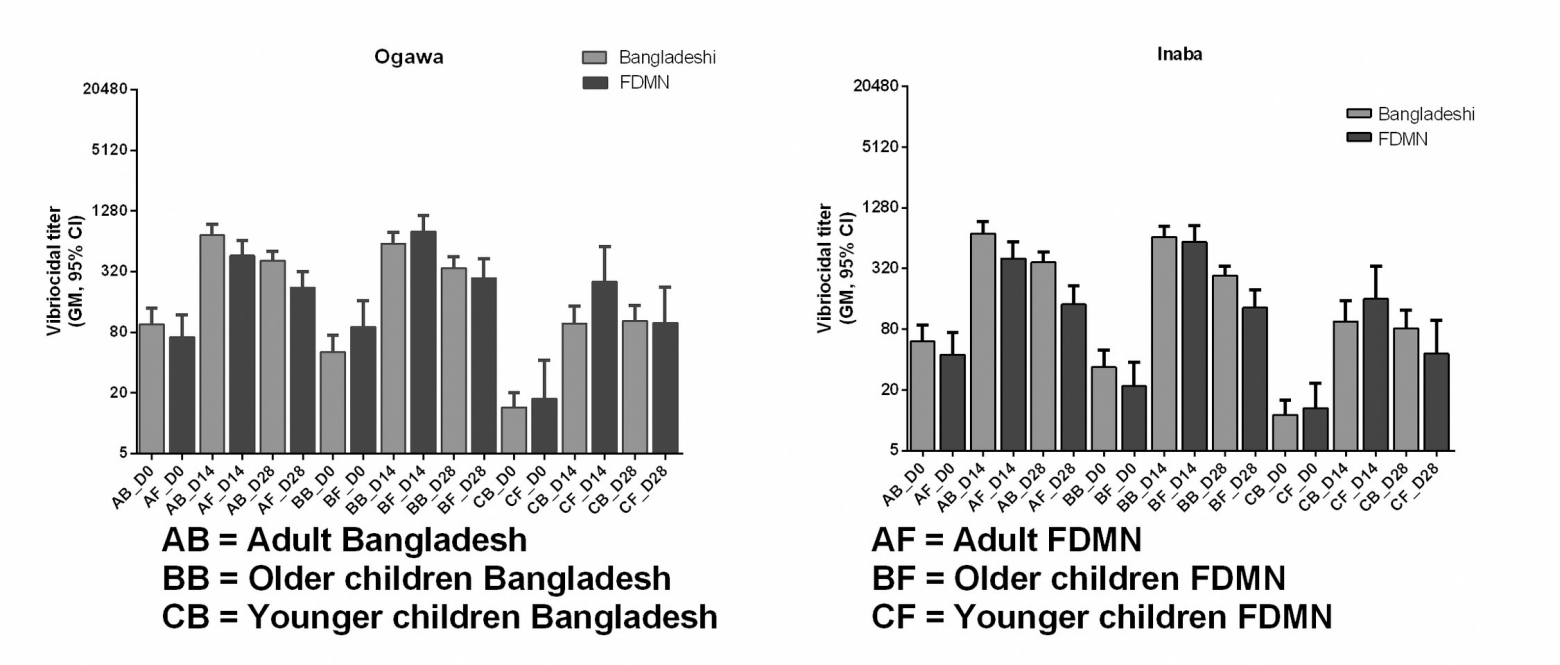
Comparison of vibriocidal antibody responses between FDMN and Bangladeshi population in adults, older children and younger children.

We compared the baseline vibriocidal antibody titer of the FDMN population with the Bangladeshi population. There were no difference in antibody titers between the two groups of participants at baseline (p = 0.07–0.08). We wanted to see whether there was any difference of response to OCV in FDMN population compared to Bangladeshi population. For Inaba serotype, differences in vibriocidal immune response between Bangladeshi vs. FDMNs are not statistically significant. Among adults GMT (95% CI) are [712 (540–937) vs. 403 (277–586)], in older children [657(517–836) vs. 586 (409–842)] and in the younger children group [96(60–152) vs. 160(78–325)]. For Ogawa serotype, immune responses are also similar among Bangladeshi and FDMN population (Fig 7 and S1 Table) in all age groups.

## Discussion

This is the first report of the immune responses to oral cholera vaccine among the FDMN participants. To facilitate ease of collection of specimens and absence of cold and refrigeration facilities on site, we wanted to see if DBS could be collected at the fragile refugee camp conditions and effectively be used for immune response studies. Thus, during sample collection we did not maintain cold chain but once brought to the field office in Cox’s Bazar, the plasma specimens were kept in the freezer at around -20°C while DBS cards were kept at 2–8°C. We also collected blood from participants by finger prick method instead of syringes so as not to aggravate any negative feeling among the displaced people to giving specimens for testing the vaccine responses. We chose the vibriocidal antibody measurement since is believed to be an indirect and surrogate marker to measure protection against cholera [11]. In addition, plasma vibriocidal antibody response was also compared among a subpopulation of the FDMN study participants. In young children, due to problems in collecting DBS only plasma antibody responses were measured.

The result of this study shows that the oral cholera vaccine Shanchol is immunogenic in adults, older children and younger children among the FDMNs. The vibriocidal antibody responses in plasma significantly increased in titers at day 14 and at day 28 in all age groups both for Ogawa and Inaba serotypes in comparison to baseline vibriocidal antibody responses. The vibriocidal response to *V*. *cholerae* O1 Inaba was 64% and to Ogawa serotype it was 64%. This is comparable to the immunogenicity studies conducted with Shanchol vaccine in Kolkata where it was 69% vs.56%; in a study of Bangladesh it was 60% and 72% respectively [18, 19] to the Inaba and Ogawa serotypes respectively. In contrast, the younger children had lower baseline vibriocidal antibody responses than adults and older children in our study. Previously, Saha *et al*. had reported the seroconversion rate and immune responses analyses from toddlers (2 to 5 years) and young children (12 to 23 months). The limitation of our study is that these age groups as well as the study days does not match with the study conducted by Saha et al. Moreover, the sample size for toddlers and young children were more than double than younger children age group in the current study [18]. Therefore, we could not compare this current data with our earlier data for younger children and older children. This is probably due to the inability of young children to respond to B cell independent carbohydrate antigens [20] and LPS is the primary antigen in the OCV. These findings are similar to the other studies conducted in other cholera endemic settings with Shanchol [21].

When we compared the immune responses between two serotypes among the study participants it is showed that significant increased of vibriocidal titre in older and younger children against Ogawa. This may due to increased exposure of *V*.*Cholerae* O1 Ogawa serotype in the Rohingya population.

Another aspect to consider is that the immune responses were of a higher magnitude after intake of the first dose of OCV among the FDMN participants as we have seen the same phenomena in earlier studies [18, 22]. We believe that this difference most likely explained by a recent and higher level of immunologic priming from previous or repeated exposure to *V*. *cholerae* O1 serotype. Also the first dose of OCV stimulates IgA antibodies in the gut, which neutralizes the vaccine antigens when given in a second dose in a short interval of 14 day [18]. Further elevation of response is therefore not seen. However, it may also be that we were not able to obtain the specimen very soon after the second dose, that is within 5–6 days of vaccination and may have missed the peak of the response as has been shown earlier in other enteric vaccines [23].

The baseline vibriocidal titers in the different age groups of vaccinees among the FDMN population were similar when compared to the responses seen in Bangladeshi healthy participants who had received Shanchol in another study (NCT02823899). When compared with results with the results of the FDMNs with other non endemic countries like Ethiopia and Haiti, it is seen that FDMN population had higher baseline vibriocidal geometric mean titer (GMT) to Ogawa (24 vs. 14 vs. 72) as well as Inaba (16 vs.11 vs. 44) serotypes seen in Ethiopia, Haiti and FDMN respectively [24, 25]. This result also suggests that the FDMN population were already exposed to *Vibrio cholerae* O1. Cholera has been reported from Yangoon in Myanmar although data from the Rakhine state of Myanmar is not available. However, it can be alluded that since Rakhine state is on the coastal area in the Bay of Bengal, which predisposes to the spread and growth of *V*.*cholerae*, it may well have been present here too causing exposure and cholera. This may be the reason for similar baseline levels of vibriocidal antibodies among the Rohingya and the Bangladeshi population.

We compared the GMT of responses in plasma obtained on day 14 and day 28 among the FDMN population with the data from the Bangladeshi population in the different age cohorts. We found that there was no difference in the level of vibriocidal antibody responses between FDMN population and Bangladeshi living in urban Dhaka (Fig 7).

In our experience, 50 μl of blood is needed to obtain a well saturated spot in DBS card and a 3.2 mm punch from this type of spot is equivalent to 1.5 μl of plasma [26]. This calculation is important to measure vibriocidal antibody titer from using eluates from DBS specimens. We had a limitation in always not being able to obtain a well saturated DBS due to varying volumes of capillary blood that may be obtained in each capillary tube [17]. We have observed that the blood collected in capillary tubes may not always be uniform. Sometimes it is possible to collect less volume of blood. This can have a negative effect on the measurement of antibody responses. If instead of 50 μl of blood, less volume say 35–40 μl is spotted on the DBS Card these may result in lower antibody measurements. We have titrated this in our laboratory and have found that a good quality blood spot is needed. If volumes are low, we observe a lower magnitude of vibriocidal titer in DBS eluates. Therefore, we were careful and attempted to use 50 μl capillary tubes and fill to the volume indicated. Based on this procedure the DBS spot were ideal for measuring vibriocidal antibody responses and which was comparison to the titer obtained using the routine venous blood collection procedure using syringes. Due to lack of facilities similar to that seen in the other humanitarian crisis, we were constrained in the FDMN camps and therefore used finger prick method for collection of blood. Parents were only willing to let their children participate in the study where needles and syringes were not used for blood collection. Another limitation in this study was that when enough blood did not flow through the capillary tubes as seen for the young children in this study, we carried out plasma separation and did not use the DBS method for analysis of immune responses. We therefore measured vibriocidal response using plasma specimens and not DBS eluates for the younger 1–5 year old children.

In the current study, we have evaluated OCV immune responses in the FDMN population by using an easy and alternate method, where we collected DBS from participants and compared immune responses with the conventional vibriocidal immune responses carried out using plasma. In a remote setting, it is not always easy to collect and process blood in adverse field settings. Therefore, DBS collection was more suitable to evaluate immune responses in settlements of Rohingyas in Cox’s Bazar. In our evaluation, we found relatively good agreement (Figs 5 and 6) between DBS and plasma vibriocidal antibody responses. We found that although the magnitude was lower when measured by the DBS method (~2 fold), it was not statistically significant (p = 0.6). Moreover, the responder frequencies were comparable when we analyzed our data from matched participants that is those who had both specimens available (DBS as well as plasma) in the participants. Based on our study, we show that that the use of DBS using precise methods is one of the most feasible ways to monitor immune responses in patients as well as vaccines [27]. Using DBS for measuring immune response to natural infection in cholera as well as to vaccines specially for seroepidemiological studies to monitor impact of implementations.

In conclusion, we show that the displaced Rohingya people in Cox’s Bazar mount good immune responses to the oral cholera vaccine and comparable responses as seen in Bangladeshi participants. The baseline titer vibriocidal antibody titer is similar responses as that seen in Bangladeshis or that seen in other cholera endemic settings. Our study results suggest that the Rakhine state in Myanmar is possibly also endemic for cholera. The oral cholera vaccine is immunogenic and contributes to the body of evidence favoring the use of OCV as a component of comprehensive cholera control. Moreover, from our study results we can say that the immunogenicity response from both DBS and plasma did not differ significantly, hence in a resource poor setting such as the refugee camps or during humanitarian crisis, DBS may be useful to collect specimen to study immunogenicity analysis. However, the collection of blood on DBS cards must be carried out with care especially in young children and a minimum of 50μl blood needs to be applied. With this caution, the DBS method can be used in refugee settings and in all age groups.

## Supporting information

S1 Table Vibriocidal antibody response in Bangladeshi population and FDMNs. (DOCX)

S1 Text Vibriocidal antibody assay. (DOC)

## References

[1] Mahmood SS, Wroe E, Fuller A, Leaning J. The Rohingya people of Myanmar: health, human rights, and identity. The Lancet. 2017;389(10081):1841–50. 10.1016/S0140-6736(16)00646-2 27916235

[2] Beyrer C, Kamarulzaman A. Ethnic cleansing in Myanmar: the Rohingya crisis and human rights. The Lancet. 2017;390(10102):1570–3. 10.1016/S0140-6736(17)32519-9 28943266

[3] ACTED, Faim AAMACL, International C, Aid C, NGOs CoD, Council DR, et al. One Year On: Meaningful progress needed to end impunity, discrimination and segregation in Rakhine State, say international agencies 2018 2018 [updated August 27; cited 2018 December 22]. Available from: https://reliefweb.int/report/myanmar/one-year-meaningful-progress-needed-end-impunity-discrimination-and-segregation.

[4] Pennington M. Bangladesh point finger at Myanmar for Rohingya 'genocide' 2018 [updated 27 September 2018; cited 2018 December 21]. Available from: https://www.foxnews.com/world/bangladesh-point-finger-at-myanmar-for-rohingya-genocide.

[5] Summers A, Humphreys A, Leidman E, Van Mil LT, Wilkinson C, Narayan A, et al Notes from the Field: Diarrhea and Acute Respiratory Infection, Oral Cholera Vaccination Coverage, and Care-Seeking Behaviors of Rohingya Refugees—Cox’s Bazar, Bangladesh, October–November 2017. Morbidity and Mortality Weekly Report. 2018;67(18):533 10.15585/mmwr.mm6718a6 29746454 PMC5944978

[6] Aung WW, Okada K, Na-Ubol M, Natakuathung W, Sandar T, Oo NAT, et al Cholera in Yangon, Myanmar, 2012–2013. Emerging Infectious Diseases. 2015;21(3):543 10.3201/eid2103.141309 25695393 PMC4344278

[7] WHO. Global Health Observatory Myanmar statistics [cited 2018 October 31]. Available from: http://apps.who.int/gho/data/node.country.country-MMR?lang=en.

[8] WHO. Weekly epidemiological record 2016 [cited 2018 October 31]. 433–40]. Available from: http://apps.who.int/iris/bitstream/handle/10665/250142/WER9138.pdf?sequence=1.

[9] Review: Rohingya influx since 1978 2017 [cited 2018 December 22]. Available from: https://www.acaps.org/sites/acaps/files/slides/files/20171211_acaps_rohingya_historical_review.pdf.

[10] Cousins S. Rohingya threatened by infectious diseases. The Lancet 2018;18(6):609–10. 10.1016/S1473-3099(18)30304-9 31272735

[11] Domman D, Chowdhury F, Khan AI, Dorman MJ, Mutreja A, Uddin MI, et al Defining endemic cholera at three levels of spatiotemporal resolution within Bangladesh. Nature genetics. 2018;50(7):951 10.1038/s41588-018-0150-8 29942084 PMC6283067

[12] Islam MT, Khan AI, Sayeed MA, Amin J, Islam K, Alam N, et al Field evaluation of a locally produced rapid diagnostic test for early detection of cholera in Bangladesh. PLoS neglected tropical diseases. 2019;13(1):e0007124 10.1371/journal.pntd.0007124 30703097 PMC6372204

[13] Qadri F, Azad AK, Flora MS, Khan AI, Islam MT, Nair GB, et al Emergency deployment of oral cholera vaccine for the Rohingya in Bangladesh. The Lancet. 2018;391(10133):1877–9. 10.1016/S0140-6736(18)30993-0 29781432

[14] Qadri F, Wierzba TF, Ali M, Chowdhury F, Khan AI, Saha A, et al Efficacy of a single-dose, inactivated oral cholera vaccine in Bangladesh. New England Journal of Medicine. 2016;374(18):1723–32. 10.1056/NEJMoa1510330 27144848

[15] Saha A, Khan A, Salma U, Jahan N, Bhuiyan TR, Chowdhury F, et al The oral cholera vaccine Shanchol^™^ when stored at elevated temperatures maintains the safety and immunogenicity profile in Bangladeshi participants. Vaccine. 2016;34(13):1551–8. 10.1016/j.vaccine.2016.02.020 26896684

[16] Qadri F, Mohi G, Hossain J, Azim T, Khan A, Salam M, et al Comparison of the vibriocidal antibody response in cholera due to Vibrio cholerae O139 Bengal with the response in cholera due to Vibrio cholerae O1. Clin Diagn Lab Immunol. 1995;2(6):685–8. 8574829 10.1128/cdli.2.6.685-688.1995 PMC170220

[17] Bhuiyan MS, Hossain M, Sharmin S, Shirin A, Khanam F, Chowdhury F, et al Assessment of disease specific immune responses in enteric diseases using dried blood spot (DBS). PloS one. 2019;14(6):e0218353 10.1371/journal.pone.0218353 31206533 PMC6578496

[18] Saha A, Chowdhury MI, Khanam F, Bhuiyan MS, Chowdhury F, Khan AI, et al Safety and immunogenicity study of a killed bivalent (O1 and O139) whole-cell oral cholera vaccine Shanchol, in Bangladeshi adults and children as young as 1 year of age. Vaccine. 2011;29(46):8285–92. 10.1016/j.vaccine.2011.08.108 21907255

[19] Kanungo S, Desai SN, Nandy RK, Bhattacharya MK, Kim DR, Sinha A, et al Flexibility of oral cholera vaccine dosing—a randomized controlled trial measuring immune responses following alternative vaccination schedules in a cholera hyper-endemic zone. PLoS neglected tropical diseases. 2015;9(3):e0003574 10.1371/journal.pntd.0003574 25764513 PMC4357440

[20] Chowdhury F, Khan AI, Harris JB, LaRocque RC, Chowdhury MI, Ryan ET, et al A comparison of clinical and immunologic features in children and older patients hospitalized with severe cholera in Bangladesh. The Pediatric infectious disease journal. 2008;27(11):986 10.1097/INF.0b013e3181783adf 18833030 PMC2749325

[21] Kanungo S, Lopez AL, Ali M, Manna B, Kim DR, Mahapatra T, et al Vibriocidal antibody responses to a bivalent killed whole-cell oral cholera vaccine in a phase III trial in Kolkata, India. PloS one. 2014;9(5):e96499 10.1371/journal.pone.0096499 24800828 PMC4011749

[22] Alam MM, Riyadh MA, Fatema K, Rahman MA, Akhtar N, Ahmed T, et al Antigen-Specific Memory B-Cell Responses in Bangladeshi Adults after One- or Two-Dose Oral Killed Cholera Vaccination and Comparison with Responses in Patients with Naturally Acquired Cholera. Clinical and Vaccine Immunology. 2011;18(5):844–50. 10.1128/CVI.00562-10 21346055 PMC3122537

[23] Akhtar M, Qadri F, Bhuiyan TR, Akter S, Rafique TA, Khan A, et al Kinetics of antibody-secreting cell and fecal IgA responses after oral cholera vaccination in different age groups in a cholera endemic country. Vaccine. 2017;35(2):321–8. 10.1016/j.vaccine.2016.11.055 27916412

[24] Desai SN, Akalu Z, Teshome S, Teferi M, Yamuah L, Kim DR, et al A randomized, placebo-controlled trial evaluating safety and immunogenicity of the killed, bivalent, whole-cell oral cholera vaccine in Ethiopia. The American journal of tropical medicine and hygiene. 2015;93(3):527–33. 10.4269/ajtmh.14-0683 26078323 PMC4559691

[25] Charles RC, Hilaire IJ, Mayo-Smith LM, Teng JE, Jerome JG, Franke MF, et al Immunogenicity of a killed bivalent (O1 and O139) whole cell oral cholera vaccine, Shanchol, in Haiti. PLoS neglected tropical diseases. 2014;8(5):e2828 10.1371/journal.pntd.0002828 24786645 PMC4006712

[26] Mei JV, Alexander JR, Adam BW, Hannon WH. Use of filter paper for the collection and analysis of human whole blood specimens. The Journal of nutrition. 2001;131(5):1631S–6S. 10.1093/jn/131.5.1631S 11340130

[27] Roberts T, Cohn J, Bonner K, Hargreaves S. Scale-up of routine viral load testing in resource-poor settings: current and future implementation challenges. Clinical Infectious Diseases. 2016;62(8):1043–8. 10.1093/cid/ciw001 26743094 PMC4803106

