# Supplementary material for: Immunogenicity of a killed bivalent whole cell oral cholera vaccine in forcibly displaced Myanmar nationals in Cox's Bazar, Bangladesh
Source: PLoS Negl Trop Dis. 2020 Mar 16;14(3):e0007989. doi: 10.1371/journal.pntd.0007989 (PMC7075546; doi:10.1371/journal.pntd.0007989)
Supplement: S1 Table — (DOCX) [file pntd.0007989.s001.docx]

**S1 Table: Vibriocidal abtibody response in Bangladeshi population and FDMNs**

| Serotype | Age Group | Bangladeshi | FDMN |
| --- | --- | --- | --- |
|  |  | GMT* ( 95% confidence interval) | |
| Inaba | Adult | 712 (540-937) | 403 (277-586) |
|  | Older Children | 657 (517-836) | 586 (409-842) |
|  | Young Children | 96 (60-152) | 160 (78-325) |
| Ogawa | Adult | 732 (571-938) | 460 (330-643) |
|  | Older Children | 606 (468-785) | 797 (554-1145) |
|  | Young Children | 97 (65-145) | 253 (119-541) |

* Geometric Mean Titer
