# Supplementary material for: Immunogenicity of a killed bivalent whole cell oral cholera vaccine in forcibly displaced Myanmar nationals in Cox's Bazar, Bangladesh
Source: PLoS Negl Trop Dis. 2020 Mar 16;14(3):e0007989. doi: 10.1371/journal.pntd.0007989 (PMC7075546; doi:10.1371/journal.pntd.0007989)
Supplement: S1 Text — (DOC) [file pntd.0007989.s002.doc]

1. Culture *V. cholerae* O1 (strain 19479 El Tor. Inaba and strain X25049 El Tor) on blood agar plates at 37^o^C overnight.
2. Inoculate a loopful of bacteria from the plates in 15 ml Brain Hear Infusion (BHI)-medium in a conical flask with cotton plug. Incubate on a shaker at 37^o^C for 3-4 hours.
3. Centrifuge the culture at 3000 rpm for 10 minutes. Throw away the supernatant, resuspend the sediment in sterile saline.
4. Recentrifuge for another 8-10 minutes. Resuspend the pellet in sterile saline.
5. Adjust bacterial concentration by spectrophotometer at 600 nm.

For *V. cholerae* O1 adjust OD at 0.3

1. Dilute heat-inactivated (56^o^C. 30 minutes) sera 2-fold in sterile saline in flat-bottom microtitre plates (Nunc, F) as follows:
2. Dispense 25 µl of cold saline in all wells except column #2.
3. Dilute test sera at 1:10 dilution and dispense in column #2. Serially dilute the sera 2-fold by using a multichannel dispenser. The dilution is accomplished by mixing the solution in column #2, aspirating 25 µl and dispensing and mixing the sample in column #3 and so on, till column #12 (this equals to 1:10240). Discard the last 25 µl from the last well on each row. Keep the plates at 4^o^C until used.
4. Add heat inactivated pool (VC01 pooled) sera in every plate as a control (row G and row H).
5. Prepare indicator (bacteria-complement-saline mixture). The composition for each plates is as follows:

**Saline Bacteria GP Complement**

*V. cholerae* O1/0139

(X25049, T19479, 134B) 2.55 ml 150µl 300 µl

Use immediately after preparation.

1. Add 25 µl of the indicator to all wells except wells in row E, F, G and H in column #1. Incubate the plate on a shaker at 37^o^C for 1 hour (50 rev/min).
2. Add 150 µl BHI/well. Incubate for another 2-3 hours at 37^o^C without shaking. Read the plates visually and spectrophotometrically. The absorbance for control wells should reach 0.20 to 0.28 at 595 nm.
3. Vibriocidal antibody titre is defined as the reciprocal of the highest serum dilutions resulting in greater than 50% OD reduction when compared to control wells without serum. An increase of titre by 4-fold between acute and convalescent sera is considered to be significant.

Serum

1:10 dilution

Complement (Guinea pig)

**Reagents and Equipment**

**Normal Saline:** 9.0 g / L NaCl (Cat No: K28549200 048, Merck, Germany)

**Blood Agar Plate:** Blood Agar Base dehydrated media + 5% sheep blood (Cat No: 245400, DIFCO)

**Brain Heart Infusion Broth:** Brain Heart Infusion dehydrated media (Cat No: 237500, BD)

**Titertek Multiskan Plus:** Biotek, EON ELISA Reader

**Spectrophotometer:** Beckman Coulter DU530

**Shaker Incubator:** Thermo Fisher Scientific MaxQ 4000

**Centrifuge:** Thermo Fisher Scientific Sorvall Legend XTR

**Incubator:** Heratherm

**Plate:** Cat No: 269620; Thermo Fisher Scientific NUNC 96F Microwell plate

**Inter assay variation:**

Titer of batch pooled sera with batch of guinea pig complement should not vary more than two-fold.

**Intra assay variation:**

Should be similar in duplicate rows. Maximum variation is 2-fold.

**Criteria for performing repeat:**

Test plate should be repeated

1. If Optical density of the indicator are <0.2 or >0.28
2. If titre of the Pooled sera (control) does not match to the optimized value.
3. If the titre of sample of duplicate row vary more than two-fold.
4. If there is any unexpected O.D. in the well of negative control.
